# Quantifying the Workability of Calcium Sulfoaluminate Cement Paste Using Time-Dependent Rheology

**DOI:** 10.3390/ma15165775

**Published:** 2022-08-21

**Authors:** Sukanta K. Mondal, Adam Welz, Carrie Clinton, Kamal Khayat, Aditya Kumar, Monday U. Okoronkwo

**Affiliations:** 1 Sustainable Materials Laboratory (SusMatLab), Missouri University of Science and Technology, Rolla, MO 65409, USA; 2 Department of Chemical & Biochemical Engineering, Missouri University of Science and Technology, Rolla, MO 65409, USA; 3 Department of Civil, Architectural and Environmental Engineering, Missouri University of Science and Technology, Rolla, MO 65409, USA; 4 Department of Materials Science and Engineering, Missouri University of Science and Technology, Rolla, MO 65409, USA

**Keywords:** calcium sulfoaluminate cement, chemical admixture, retarder, rheology, workability

## Abstract

Poor workability is a common feature of calcium sulfoaluminate (CSA) cement paste. Multiple chemical admixtures, such as set retarders and dispersants, are frequently employed to improve the workability and delay the setting of CSA cement paste. A quantitative assessment of the compatibility, efficiency, and the effects of the admixtures on cement paste workability is critical for the design of an appropriate paste formulation and admixture proportioning. Very limited studies are available on the quantitative rheology-based method for evaluating the workability of calcium sulfoaluminate cement pastes. This study presents a novel and robust time-dependent rheological method for quantifying the workability of CSA cement pastes modified with the incorporation of citric acid as a set retarder and a polycarboxylate ether (PCE)-based superplasticizer as a dispersant. The yield stress is measured as a function of time, and the resulting curve is applied to quantify three specific workability parameters: (i) the rate at which the paste loses flowability, (ii) the time limit for paste placement or pumping, marking the onset of acceleration to initial setting, and (iii) the rate at which the paste accelerates to final setting. The results of the tested CSA systems show that the rate of the loss of flowability and the rate of hardening decrease monotonously, while the time limit for casting decreases linearly with the increase in citric acid concentration. The dosage rate of PCE has a relatively small effect on the quantified workability parameters, partly due to the competitive adsorption of citrate ions. The method demonstrated here can characterize the interaction or co-influence of multiple admixtures on early-age properties of the cement paste, thus providing a quantitative rheological protocol for determining the workability and a novel approach to material selection and mixture design.

## 1. Introduction

Calcium sulfoaluminate (CSA) cement is one of the widely researched, low-energy, low-carbon alternatives to conventional Portland cement (PC) [1,2]. The production of CSA cement emits 60% less CO_2_ than that of PC—the latter contributes to about 8% of the global CO_2_ emissions [3,4,5]. The lower emission potential of CSA cement is primarily due to the lower calcareous raw material (i.e., limestone) content required to produce the main cementing phase in CSA clinker, ye’elimite (Ca_4_Al_6_SO_16_), that releases ~0.22 t CO_2_/t compared to the main cementing phase of PC, alite (Ca_3_SiO_5_), that generates ~0.58 t CO_2_/t [6]. Additionally, a lower processing kiln temperature of 1250 °C is required for CSA cement production, in contrast to 1450 °C needed for PC production, leading to additional fuel-derived CO_2_ emission savings and a beneficial energy economy [2,6]. CSA cement is also attractive due to its unique characteristics in specialty applications for rapid setting and expansion-shrinkage compensating paste formulations, although unanswered questions remain regarding the long-term durability of CSA cement [7]. 

Poor early-age workability arguably remains one of the major challenges that limit the general applicability of calcium sulfoaluminate (CSA) cements in conventional building construction, wherein the poor workability is a trade-off on the quick setting and high early strength development of CSA paste. Two approaches can be employed to control CSA workability, namely: (i) reaction rate control by regulation of the clinker chemistry or the proportions of the ye’elimite, belite, and anhydrite or gypsum in the cement powder [8,9,10,11], and (ii) use of chemical admixtures to control the reaction rate and/or improve the dispersion and fluidity. The latter is the most common approach in the field as it is easy to apply on-site [12,13]. Organic acid and sugars are often used to effectively delay the CSA rapid hydration reaction, while polymer dispersants (i.e., superplasticizers) are employed to enhance dispersion and fluidity [14,15,16]. In practice, multiple admixtures are frequently employed simultaneously to improve the workability of CSA cement. For example, a combination of superplasticizer and set retarder can be used to improve the flowability of the CSA system [17,18,19]. The superplasticizer provides electrostatic dispersion and steric hinderance, while the set retarder delays the hydration of cement particles and the formation of hydration products, thus enhancing the workability retention [19]. For such applications, where multiple chemical admixtures are incorporated in CSA cement paste, it is crucial to evaluate the compatibility and co-influence of the admixtures on the workability and its variation at an early age [20,21,22]. This is especially important for advanced paste formulations, such as those for 3D printing and oil well cementing applications [23,24,25].

The slump test is one of the common methods used to evaluate the workability and consistency of fresh concrete and mortar [13]. Although the method provides some indirect flow properties, it is limited [26] and inadequate for time-dependent characterization of the workability and flow dynamics of advanced cement-based mixture designs [27]. A wide range of other approaches have been reported for measuring the workability of cement-based materials, which are classifiable under five major categories: confined flow, free flow, vibration, moving object, and rheology-based test methods [28,29]. Amongst these methods, the rheology-based approaches feature the best technical versatility [29]. 

Thus, advanced assessment of the workability of fresh cement-based materials often involves the measurement of basic physical quantities of the fluid property, for example, yield stress and viscosity [30]. These rheological parameters can be used to estimate the flowability and provide insight on the workability. However, most of the available rheology-based studies of CSA cement materials do not provide quantitative estimation of the workability [31,32]. Various analytical models have been applied to describe the rheological behavior of cement paste, including the Bingham, Casson, Herschel–Bulkley, and De Kee models, etc., which are mainly used to extract yield stress and viscosity [33,34]. However, these models have not been used to extract quantitative workability parameters that examine the various regimes of the hydration reaction progress to quantifying the flowability retention, placement time, and hardening rate, as presented in the present study. 

There are other relevant studies, such as those by Roussel [35] describing the transient and steady flow behavior of cement paste, the bi-linear model by Kruger et al. [36,37], and the non-linear model by Lecompte et al. [38]. However, these models mainly focused on estimating the buildability and shape retention of 3D-printed cement pastes, rather than the quantification of the comprehensive workability of the pastes. In the present study, a unique quantitative approach is presented for determining the workability of CSA cement using a time-dependent rheological method. The approach quantifies the influence of multiple admixtures on the critical early-age hydration regimes to determine specific workability metrics vis-à-vis flowability retention, placement limit, yield stress growth rate constant, and hardening rate, as a function of admixture dosage, thereby providing a novel approach to evaluating the workability of advanced CSA cement paste formulations.

## 2. Materials and Experimental Methods

### 2.1. Materials

**Figure 1:**
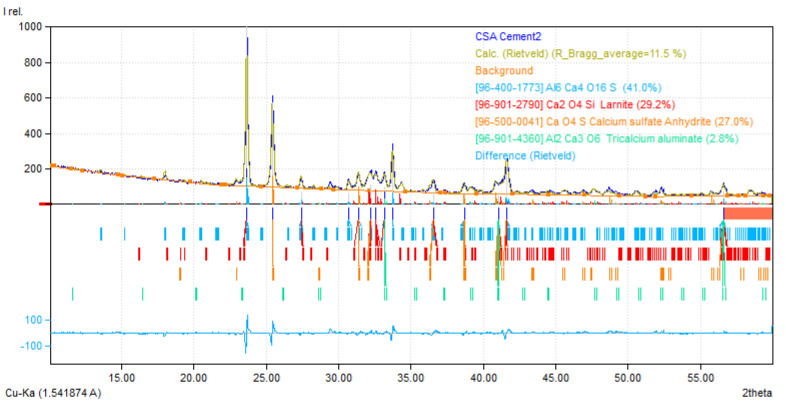
Rietveld refinement of the X-ray powder diffraction pattern of the CSA cement, showing percentages of the constituent minerals. The reference patterns for the identified phases, ye’elimite [39], belite (larnite [40]), anhydrite [41], and aluminate [42], are from the Crystallography Open Database (COD).

A commercial calcium sulfoaluminate cement received from Buzzi Unicem USA was used. The oxide composition of the cement, analyzed by energy-dispersive X-ray fluorescence (EDXRF; Oxford X-Supreme 8000, Abingdon, UK) and verified by X-ray photoemission spectroscopy (XPS; Thermoscientific Nexsa Surface Analysis, Waltham, MA, USA), is shown in Table 1. The X-ray diffraction (XRD) pattern of the CSA cement, obtained by a PANalytical X’Pert Pro multipurpose diffractometer with a 2θ configuration using CuKα (λ = 1.540 Å) radiation, is shown in Figure 1. The mineral compositions of the materials were quantified by Rietveld refinement of the X-ray diffraction pattern [31], with the aid of the FullProf Suite integrated into the Match!—phase analysis by powder diffraction software. The quantitative phase analysis shows that the CSA cement is a belite-ye’elimite cement composed of 41.0% ye’elimite (C_4_A_3_S), 29.2% belite (C_2_S), 27.0% anhydrite (C$\bar{S}$), and minor 2.8% aluminate (C_3_A) constituents (Figure 1). A reagent grade, powder citric acid (CA) additive (citric acid monohydrate, 99.5%) was used as a set retarder, and a commercially available ASTM C494 Type A and F and ASTM C1017 Type I compliant polycarboxylate ether (PCE)-based superplasticizer was used to improve dispersion. The authors acknowledge the expectation that potential competitive adsorption may limit the effectiveness of PCE in the system, but it is anticipated that the presented rheological method may provide some insight on the complex interactions.

### 2.2. Mixing Protocol

Nine CSA cement paste specimens were prepared with three different dosage rates of the PCE (0%, 0.1%, and 0.5% dry mass of PCE by mass of cement) and three different contents of citric acid (1%, 2%, and 4% dry mass of CA by mass of cement). These dosage rates were chosen in the range commonly reported in the literature [16,17,43]. It was difficult to continue the rheology experiments without any CA past 5 min given the quick setting of the mixture. Deionized water (DI) was used in preparing the cement paste, maintaining the water/cement mass ratio (w/c) as 0.5. The water content of the PCE and hydration water of the powder citric acid (CA) were accounted for as part of the total water.

The target mixture proportions of solids (CA and CSA cement) were first homogenized before mixing with liquids (a mixture of an appropriate quantity of water and PCE) to prepare the paste. In every paste, 10 g of cement was used in a 250 mL plastic beaker and mixed with a four-blade, impeller-type overhead stirrer (RW 20 Digital, IKA) for one minute at 1000 rpm. The mixed slurry was immediately loaded onto the rheometer for characterization. The same mixing protocol was used for the specimen used for the Vicat set time test, with a relatively larger quantity of materials required.

### 2.3. Parallel Plate Rheometry

The yield stress development of the fresh cementitious pastes was measured as a function of time, with a stress-controlled shear rheometer (DHR-2, TA Instruments) utilized in a parallel plate alignment using 40 mm-diameter top and bottom plates. The surfaces of the plates were crosshatched (serrated) to reduce slippage and ensure a uniform cement particle concentration near the plates [44]. The instrument and the geometry were calibrated each time before starting any run. The cement pastes were contained in a fixed geometry gap of 700 µm between the top and bottom plates. The temperature during the measurements was maintained at 25 °C by a Peltier plate connected to the bottom geometry. A solvent trap on the top plate was filled with deionized (DI) water to maintain constant humidity around the pastes during measurement. 

**Figure 2:**
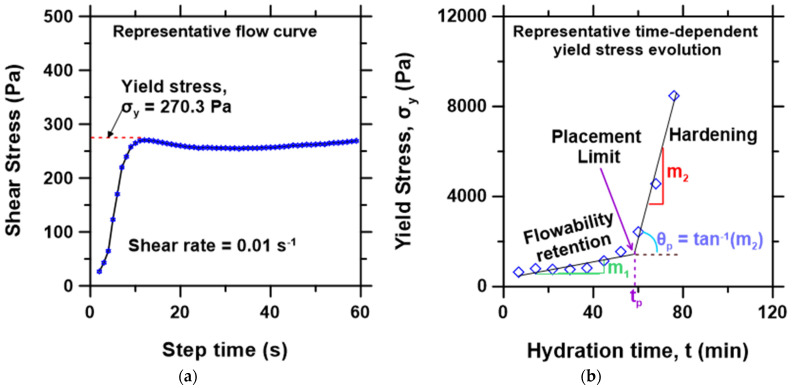
(**a**) Representative flow curve for a paste showing how yield stress (**σ_y_**) was determined. (**b**) Representative time-dependent yield stress evolution of CSA paste, showing the flowability retention regime with slope $m_{1}$, the placement limit $t_{p}$, acceleration angle $\theta_{p}$, and the hardening regime with slope $m_{2}$.

The rheology measurements were started at ~5 min, from the time of cement’s contact with the mixing water (liquid containing water and PCE), i.e., the total time for mixing and setting up the paste sample on the rheometer. The pastes were first pre-sheared at 0.1 s^−1^ or 30 s, followed by a 30 s rest, and then sheared at a very low fixed shear rate of 0.01 s^−1^ for 180 s. The shear stress at which the paste began to flow (i.e., the transition from elastic deformation to plastic flow) during the fixed shear strain rate of 0.01 s^−1^ was used to determine the “yield stress”, as exemplified in Figure 2. The peak shear stress is a realistic approximation of static yield stress [45,46]. This method is commonly referred to as the stress growth method, and the basis for its adoption in the present study is discussed in detail in Section 3.5. The above measurement steps were repeated at intervals of 3 min on the same sample to track the time-dependent evolution of yield stress of the CSA pastes until setting or when the pastes could no longer be sheared by the rheometer. Figure 2a displays a representative flow curve at a shear rate of 0.01 s^−1^ for a paste showing the determination of yield stress, and Figure 2b shows the time-dependent yield stress evolution curve, showing two linear regimes with slopes $m_{1}$ and $m_{2}$, representing the rate of loss of the paste’s flowability and the rate of hardening, respectively. The time, $t_{p}$, which marks the point of transition from the flowability regime to the rapid hardening regime represents the placement time, i.e., the time at which rapid loss of flowability sets in as the paste accelerates to setting, such that at $t_{p}$ the mixture is unsuitable to be placed but remains finishable [47]. These parameters were analyzed to examine the co-influence of the admixtures on the CSA cement paste workability, as presented in the Results and Discussion Section. It is worth noting that similar curves to the one shown in Figure 2b can be produced by measuring time-dependent dynamic yield stress or viscosity evolution, but the present study focuses on static yield for simplicity and its relevance to placement and 3D printing of cement paste [48,49,50].

### 2.4. Vicat Set Time

The time of initial set of the CSA cement pastes was also measured using a manual Vicat apparatus according to ASTM C191 [51]. The Vicat needle penetration test was carried out every 3 min. The time was counted from the mixing of CSA cement with water and admixtures. The Vicat initial set time is compared with the placement limit, $t_{p}$, from the rheological experiments.

## 3. Results and Discussion

### 3.1. Time-Dependent Yield Stress Evolution

**Figure 3:**
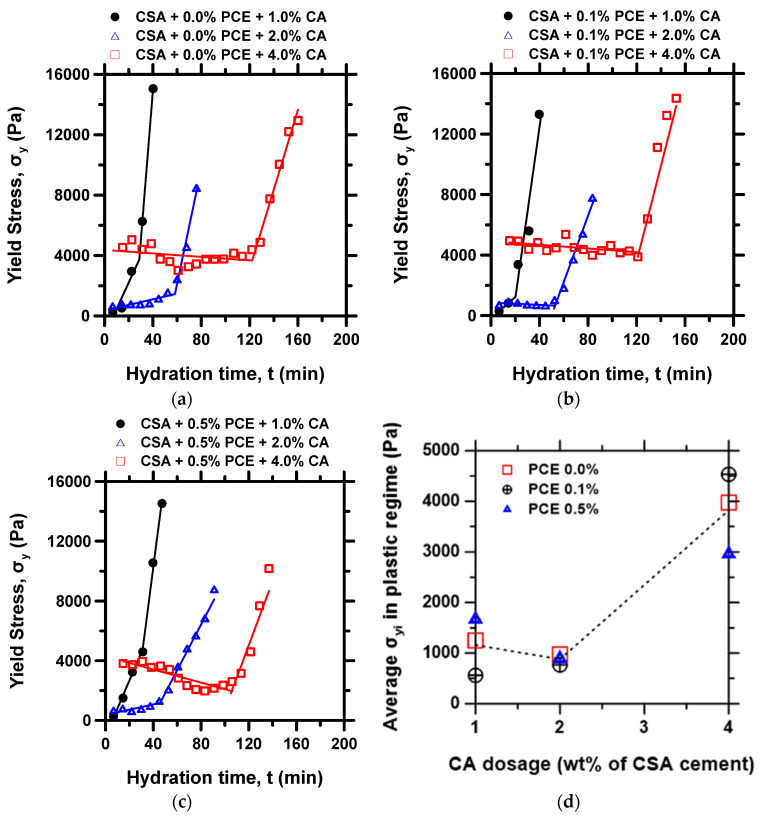
Time-dependent yield stress evolution for CSA cement pastes containing (**a**) 0% PCE, (**b**) 0.1% PCE, (**c**) 0.5% PCE, and (**d**) average yield stress at the flowability regime as a function of admixture dosage.

The time-dependent evolution of yield stress for the nine investigated mixtures is presented in Figure 3a–c as a function of hydration time, showing the broad effect of the dosage rates of PCE and CA. As expected, the duration of the flowability regime increases with the increasing content of the CA, while the dosage of the PCE does not have any observable influence on that duration. This dominant influence of CA is due to its retarding effect on hydration of the CSA compared to the PCE, which often shows a negligible retarding effect on the CSA cement reaction [14]. Interestingly, the cement paste containing 4% CA showed a significantly higher initial yield stress value than those with a lower CA content. Figure 3d displays a graph of the average yield stress (σ_yi_) determined in the flowability regime as a function of CA content. A sharp increase of σ_yi_ is noted for mixtures made with more than 2% of CA, reaching a 4-fold increase at 4% CA. Previous studies indicate that citrate anions can adsorb to positively charged cement particles, creating a particle bridging bond between interacting particles at certain pH levels [52]. Thus, an increase in CA content leads to a stronger attractive force between the cement particles, thereby increasing the yield stress. In the investigated CSA mixtures, this effect appears to increase rapidly after a CA content of 2% (Figure 3d). Therefore, while 4%CA may be beneficial for increased retardation or a prolonged flowability time of about 120 min, the trade-off is a higher initial yield stress, which implies high energy to initiate cement paste flow. Hence, in defining workability, all aspects of the yield stress curve need to be critically investigated to determine the appropriate mixture design for a given application. 

The effect of the multiple admixtures on the hardening regime is not as distinguishable by visual observation of Figure 3a–c, as all the pastes feature a steep rise in yield stress immediately following the loss of flowability. To better understand the co-influence of the PCE and CA admixtures on the rheological behavior of the CSA cement pastes, the three workability metrics (i.e., rate of loss of flowability, placement limit, and hardening rate, illustrated in Figure 2b) were determined from the time-dependent yield stress evolution curves of Figure 3a–c, and discussed in the following sub-sections.

### 3.2. Flowability Retention Metrics

**Figure 4:**
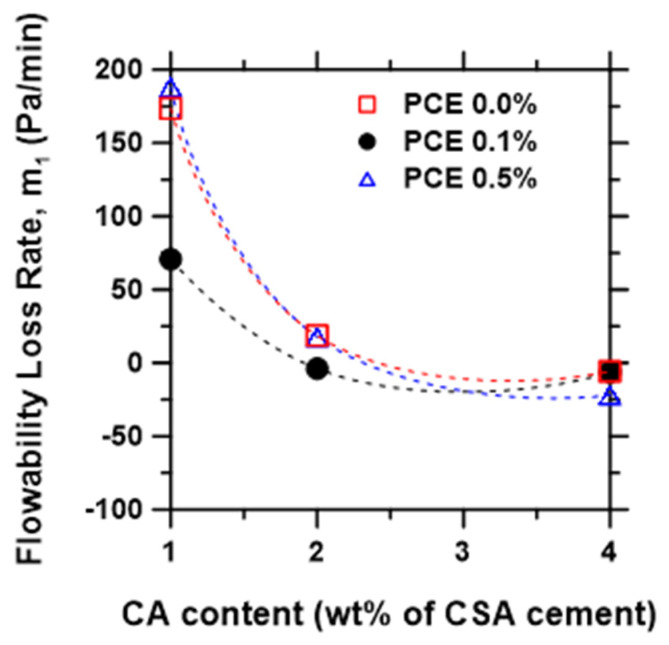
Rate of loss of flowability, $m_{1}$, of CSA cement mixtures as a function of admixture dosage.

The slope of the flowability regime of the time-dependent yield stress evolution curve (Figure 2b) was used to examine the effect of CA and PCE contents on workability retention. As shown in Figure 4, the slope $m_{1}$, which represents the rate of the loss of flowability, decreases monotonously with increasing CA content. The PCE dosage, on the other hand, shows a lesser influence, impacting a slight increase in flowability retention (i.e., decrease in the rate of the loss of flowability) at 0.1% PCE. This effect shrunk with the increasing CA content, which is attributed to competitive adsorption of the citrate over PCE [53,54,55,56]. The result suggests that PCE and CA show little or no synergy in improving retention of flowability of the CSA cement pastes, due to the PCE–CA competitive adsorption phenomenon. This is in agreement with literature data wherein PCE is reported to display higher synergy with sodium borate set retarder in improving the flowability of CSA cement pastes compared to CA [18,57]. 

It is widely reported that the performance of PCE-based superplasticizers can be perturbed by various anionic species, leading to a loss of the dispersive effect of PCE due to competitive adsorption [58]. For example, hydroxides [59,60], sulfate ions [61], citrates and tartrates [56], and other polymeric admixtures [62] are among the chemical species reported to compete for the solid surface with superplasticizers.

Plank and Winter [56] showed that in the presence of citrate, PCE adsorption dropped to less than 10% of dosage added, leading to a complete loss of flowability of the paste, whereas in the presence of tartrate, PCE adsorption was reduced but remained high enough to still provide good flowability. The study revealed a direct correlation between the admixtures’ adsorption behavior and their anionic charge density. Thus, admixtures with a higher anionic charge density show higher affinity to the binder surface and hence adsorb preferentially. 

In general, when several admixtures are present, molecules with a lower anionic charge density will adsorb only if there are any remaining adsorption surfaces after the adsorption of the molecules with a higher anionic charge density has completed. Thus, given that the anionic charge density of citrate is higher than PCE due to the comparatively smaller molecular size of the citrate ion compared to the polycarboxylate anion, the CA dosage in the present study adsorbed preferentially, thereby leaving limited surfaces available for the PCE adsorption, and hence the observed limited dispersive effect of the PCE. 

### 3.3. Placement Limit Metrics

**Figure 5:**
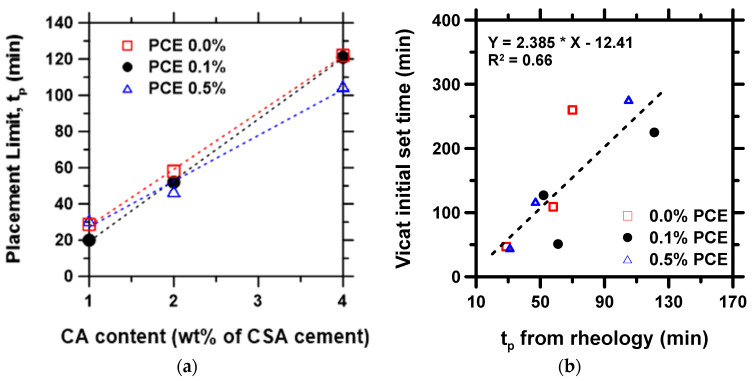
Placement time, $t_{p}$, of CSA cement paste: (**a**) evolution of $t_{p}$ as a function of admixture dosage, and (**b**) variation in initial time of set with rheology-based $t_{p}$.

The time at which the yield stress evolution curve transitions from the flowability regime to the hardening regime, defined as the placement limit, $t_{p}$, was determined, as shown in Figure 2b. The results from all the pastes were plotted as a function of CA content, as shown in Figure 5. As seen in Figure 5a, $t_{p}$ increases linearly with increasing CA, whereas the increase in the PCE dosage has a negligible effect on $t_{p}$. The result is expected, given that the CA can inhibit ettringite formation in CSA systems leading to a significant retardation of the hydration reaction [63]. On the other hand, PCE can adsorb onto cement particles, causing mainly a dispersion effect via the provision of steric hindrance and electrostatic repulsion with minimal retardation effect [64,65]. Figure 5b shows that $t_{p}$ correlates somewhat linearly with the initial time of set measured with the Vicat apparatus. While both parameters ($t_{p}$ and Vicat set time) are different, the linear relationship, although not as strong compared to similar data for Portland cement systems [47], indicates that the study of the early-age rheological behavior of CSA can inform some aspects of setting, in addition to providing a means to assess the co-influence of chemical admixtures in CSA cement pastes for optimizing the workability.

### 3.4. Hardening Rate Metrics

**Figure 6:**
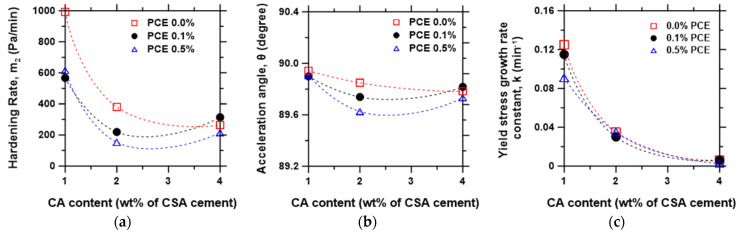
The hardening rate, $m_{2}$, of the CSA cement pastes: (**a**) the evolution of $m_{2}\;$ as a function of admixture dosage, (**b**) the evolution of acceleration angle, $\theta_{p}$, and (**c**) Yield stress growth rate constant k.

The hardening rate ($m_{2}$), which is the slope of the regime with a steep increase in yield stress as the paste rapidly accelerates to setting, was extracted from the time-dependent yield stress evolution curve, as shown in Figure 2b. The $m_{2}$ indicates a monotonous decrease with increasing CA content (Figure 6a), similar to the trend observed for the rate of loss of flowability (Figure 4). However, there is slightly more influence of PCE dosage on $m_{2}$ than $m_{1}$, where the hardening rate decreases with the increasing PCE dosage. An alternative outlook on the hardening regime is through the acceleration angle, $\theta_{p}=\tan^{-1}m_{2}$ (Figure 2b and Figure 6b). As seen in Figure 6a,b, the $m_{2}$ and $\theta_{p}$ show similar trends; however, the $m_{2}$ curve features a steeper decline, especially between 1% and 2% CA content. This result suggests that paste mixtures with a lower CA content featuring high $m_{2}$ and high $\theta_{p}$ would be more suitable for 3D printing, while the opposite is the case for pumping applications.

The exponential growth model $\sigma_{y_{t}}\;=\;\sigma_{y_{0}}e^{kt}$ was applied to the yield stress data, where $\sigma_{y_{t}}$ is the yield stress at time *t*, $\sigma_{y_{0}}$ is the initial yield stress first measured after mixing, and k is the yield stress growth rate constant. The result is presented in Figure 6c, showing that the yield stress decreases exponentially with the increasing CA content without a noticeable effect of the PCE dosage. Overall, it can be deduced that the CSA mixture with 2% CA and 0.1% PCE featuring low average yield stress in the flowability regime ($\sigma_{y_{i}}$), low rate of flowability loss ($m_{1}$), moderate placement limit ($t_{p}$), and hardening rate ($m_{2}$) presents the best workability performance. However, as the workability score is application-dependent, the above selection may be unfavorable for certain applications. For instance, in 3D printing of cement-based materials, where a layered deposition approach is followed, a paste with a high yield stress growth rate (k), characterized by a high rate of flowability loss ($m_{1}$), and a high hardening rate ($m_{2}$) would be desirable; in contrast, for oil well cementing or extended pumping operations, a lower yield stress growth and a low rate of flowability loss would be more suitable. Thus, this technique can be used as a novelty approach to the selection of materials and mixture design for specific applications.

### 3.5. Reproducibility, Effect of Geometries, and Test Parameter Selection

This section illustrates the extent of variability of the measured rheology-based workability metrics with respect to different procedures and geometries that were screened prior to the selection of the stress growth static yield stress procedure presented in the preceding sections. To assess the methods, triplicate measurements were performed for time-dependent (1) static yield stress (i.e., using the stress growth method, with a fixed shear rate), and (2) dynamic yield stress (i.e., using Bingham model fitting of the shear stress vs. shear rate curve). These methods were tested with two geometries: (i) parallel plate geometry with crosshatched surface, and (ii) vane rotor and cylinder cup geometry. One of the nine cement paste specimens prepared in Section 2 (mix proportion: CSA + 0.1% PCE + 2% CA (w/c = 0.5)) was selected to demonstrate the reproducibility and applicability of each method. Similar to the procedure described in Section 2.3, Section 2.4, Section 3, Section 3.1, Section 3.2, Section 3.3 and Section 3.4, a temperature of 25 °C was maintained for all tests in this section.

#### 3.5.1. Static Yield Stress Tests

(i) Parallel plate geometry (crosshatched surface to reduce slippage), and (ii) vane rotor and cylinder cup geometry. 

First, triplicate measurements were performed using the stress growth method described in Section 2.3, with a 700 and a 1000 µm gap between the parallel plates, to demonstrate the effect of the geometry gap. Then, the experiment was repeated with a vane rotor and cylinder cup geometry, to understand the effect of geometry. The diameter and length of the vane rotor bob was 28 and 42 mm, while the inner diameter of the cylindrical cup was 30.33 mm. The operating gap was maintained at 4000 μm. Note that the gap is not comparable to the geometry gap of 700 or 1000 μm used for the parallel plate geometry, because the shearing effect in the cup-vane system is radially distributed. About 90 g of CSA cement was needed to prepare sufficient material for this geometry (9 times the amount required for parallel geometry). Third, the test with the parallel plate geometry gap of 700 µm was repeated, but this time a larger amount of the cement paste was prepared and stored in a sealed beaker under a 95% relative humidity, such that the paste was re-mixed ex situ with the IKA four-blade overhead mixer at 250 rpm for 30 s to break any false structure formation prior to loading fresh sample for the second step measurement, and the subsequent step intervals until setting. This was to understand the effects of mixing ex situ compared to the in situ pre-shear technique described in Section 2.3, and to help verify suspension integrity or potential sedimentation of the paste during the in situ measurement. 

**Figure 7:**
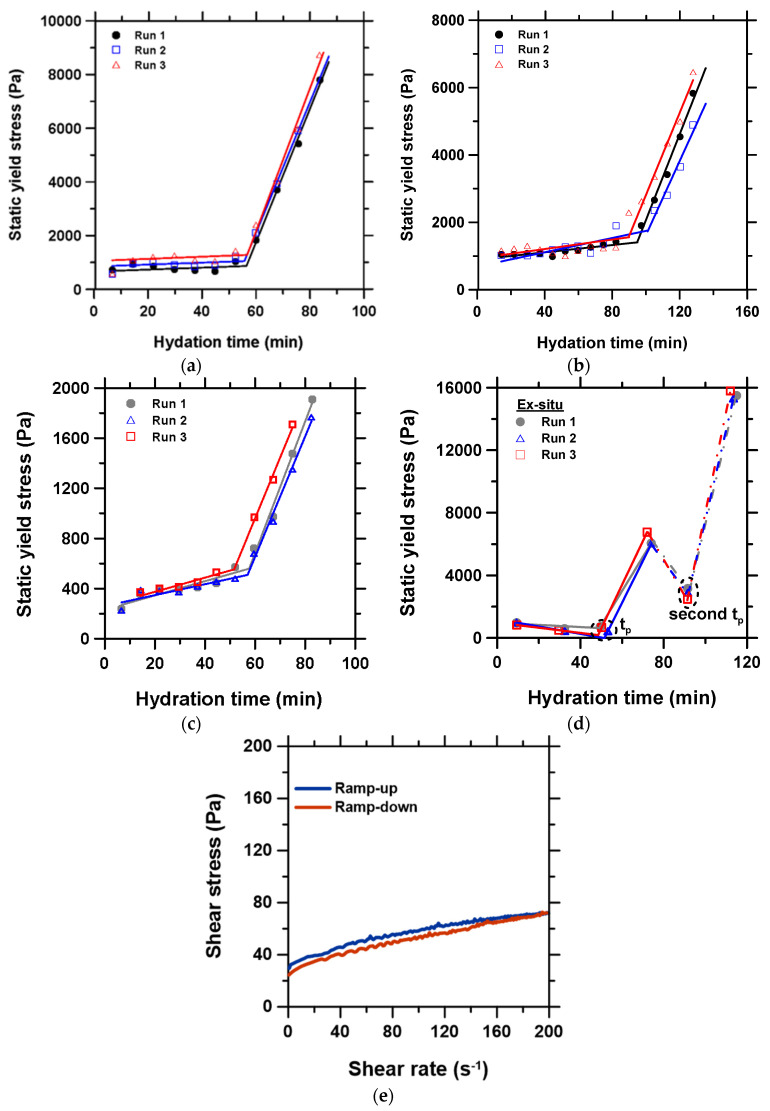
Time-dependent static yield stress evolution obtained with: (**a**) parallel plate geometry gap of 700 µm, (**b**) parallel plate geometry gap of 1000 µm, (**c**) vane rotor and cylinder cup geometry, (**d**) parallel plate geometry gap of 700 µm accompanied with ex situ re-mixing of pastes at 250 rpm prior to loading sample for each subsequent interval measurement, and (**e**) representative shear stress—shear rate curve for the ramp-up and -down between 0 and 200 s^−1^, showing a minimal hysteresis loop below the shear rate of 160 s^−1^.

Figure 7a–d show the static yield stress evolution for each triplicate test, and Table 2 presents the workability metrics, $m_{1}$**_,_** $m_{2}$, $t_{p}$, and $\theta_{p}$, obtained from the tests and their respective standard deviations. The static yield stress results indicate that the measured quantities were reasonably reproducible, wherein the relative standard errors [(std. deviation of mean/mean)×100] for each of the parameters were mostly ≤5%. The value of the $\theta_{p}$ was the most consistent across all the static yield stress methods. It was observed that increasing the parallel plate geometry gap from 700 to 1000 µm led to a slight increase in $m_{1}$, a slight decrease in $m_{2}$, and a notable increase in $t_{p}$. A higher propensity for error due to the slippage phenomenon near the $t_{p}$ point was observed for the 1000 µm compared to the 700 µm geometry gap. Thus, the overall reproducibility was better for the 700 µm than for the 1000 µm geometry gap, as seen in Figure 7a,b. Note that both gaps were within the commonly recommended range, >10× the particle size [66] (average D50 of the CSA cement used is 1 µm). 

As seen in Figure 7d, the ex situ mixing method exhibited two $t_{p}$ points. The first $t_{p}$ value corresponds to the value obtained with both the in situ parallel plate geometry gap of 700 µm and the in situ vane rotor and cylinder geometry. The flowability regime of the yield stress curve displays a negative slope, which suggests that the ex situ mixing had improved the action of the dispersant with time, by breaking any initial flocs. Additionally, the ex situ mixing at 250 rpm is believed to have disrupted the initial onset of structuration, leading to a quick drop in yield stress and eventual re-establishment of the structuration at the second $t_{p}$ prior to acceleration to the final setting. However, in general, the first hardening regime, $m_{2}$, following the first $t_{p}$ point clearly agrees with the test results from the in situ parallel plate geometry with the 700 µm gap. It can therefore be concluded that there was no sedimentation issue in the in situ parallel tests.

Consistent with recommendations in the literature [67,68], a low shear rate of 0.01 s^−1^ as described in Section 2.3 was adopted for the parallel plate-based static yield stress tests. Figure 7e shows a representative behavior of the paste in response to a shear rate or 0–200 s^−1^, showing a minimal hysteresis loop between 0 and 160 s^−1^. The hysteresis loop appeared to decrease slightly with the increasing shear rate and disappeared at about 160 s^−1^. However, a low shear rate is favored for the parallel plate geometry in the present study because a high shear rate can break the cement structure even close to setting [68], and the cement paste tends to bleed out of the parallel plate confinement at high shear, creating voids and erroneous data. However, it is noteworthy that the measured rheological values are generally dependent on the applied shear stress and strain [45]. To better understand the effect of a variable shear rate, dynamic tests are investigated in the following sub-section.

#### 3.5.2. Dynamic Yield Stress and Plastic Viscosity Tests

(i) Parallel plate geometry and (ii) vane rotor and cylinder cup geometry:

It is known that both static yield stress and dynamic yield stress are important for different field applications [69]. For example, the dynamic yield stress of cement pastes will provide useful information regarding cement flow behavior in pumping applications, whereas the static yield stress can provide helpful data to inform paste stability [70] and 3D printability of cement pastes [23,24,25], etc. To understand the differences and illustrate the applicability of the dynamic yield stress and plastic viscosity evolution to estimating workability metrics described in the preceding sections, we investigated the traditional fitting of the Bingham model to the shear stress vs. shear rate curve [66]. The selected paste sample (CSA + 0.1% PCE + 2% CA (w/c = 0.5)) was characterized by the Bingham model-fitting method using both the parallel plate geometry and the vane rotor and cylinder geometry. A variable shear rate was imposed on the paste with a ramp-up of 0–200 s^−1^ that was immediately followed by a ramp-down after a short rest of 5 s. The ramp-up served as pre-shear, while the dynamic yield stresses and the plastic viscosities were determined from the ramp-down utilizing the Bingham model, $\sigma^{*}\;=\;\sigma_{y}\;+\;\dot{\gamma }\cdot \;{µ}_{pl}$, where $\sigma^{*}$ is the shear stress in a step (variable), $\sigma_{y}$ is the yield stress, $\dot{\gamma }$ is the shear rate (0–200 s^−1^), and ${µ}_{pl}$ is the plastic viscosity (i.e., slope of the straight-line path). 

**Figure 8:**
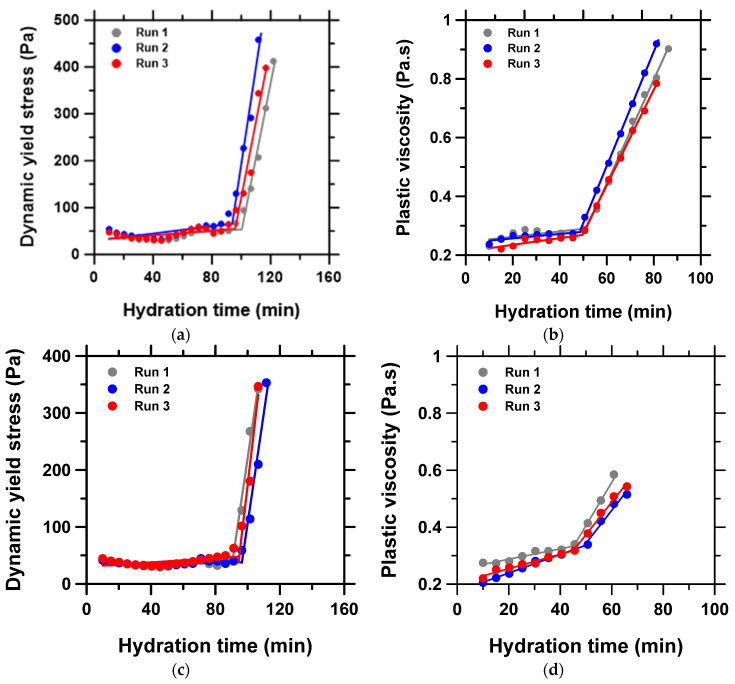
Time-dependent dynamic yield stress and plastic viscosity evolution obtained with: (**a**,**b**) parallel plate geometry with a gap of 700 µm, and (**c**,**d**) vane rotor and cylinder cup geometry, respectively.

The evolution of the result of the triplicate measurement of the time-dependent dynamic yield stress and the plastic viscosity are presented in Figure 8, and workability parameters in Table 3. As expected, the dynamic yield stress was significantly lower than the static yield stress discussed previously. Interestingly, the data from the parallel plate geometry of 700 µm and the vane rotor and cylinder cup geometry were very similar, suggesting comparable suitability of both methods. However, the values of all the workability parameters obtained with time-dependent plastic viscosity were generally lower than the corresponding values obtained with dynamic yield stress evolution, except for the $\theta_{p}$ parameter. In general, the dynamic yield stress from vane rotor and cylinder geometry showed slightly better reproducibility, while the plastic viscosity from the parallel plate geometry showed better reproducibility. Additionally, it is noted that deviations in the trends were much more pronounced as the hydrating paste transitioned from the initial dormant state to the rapid acceleration stage of the hydration. This is reflected in the magnitude of the standard deviation on $m_{2}$ compared to $m_{1}$. There was also less reproducibility for plastic viscosity compared to the dynamic yield stress measurement.

In summary, the in situ static yield stress procedure with a parallel plate geometry gap of 700 µm was adopted for the main demonstrations discussed in Section 2.3, Section 2.4, Section 3, Section 3.1, Section 3.2, Section 3.3 and Section 3.4, for the simplicity, requirement of small material, reasonable reproducibility, and relevance of static yield stress to 3D printing of cement slurry. However, any of the above-discussed rheological methods and geometry can be used to produce “self-consistent” workability metrics for assessing cement mixtures. It is also possible to use oscillatory rheology (not covered in the present study), wherein the time-dependent evolution of the storage modulus and loss modulus can be used to develop the workability metrics. However, it is well-known in the literature that a small change in rheology procedures and test parameters, including mixing protocols and shear history of the pastes, can affect the absolute value of the measured rheological quantities [71,72,73,74]. Hence, a selection of a favorable protocol must be performed after screening for any specific materials being studied and the selected parameters must be carefully maintained for all mix proportions under comparison to reduce errors. Additionally, caution must be applied when using the measured workability metrics as absolute number values, instead they should be used as a relative measure to assess mix proportions.

## 4. Conclusions

A rheology-based method was employed to develop quantitative metrics for assessing the workability of CSA cement pastes prepared with citric acid set retarder (CA) and polycarboxylate ether-based superplasticizer (PCE). The method utilizes a time-dependent yield stress evolution curve to quantify specific workability parameters that describe the various regimes of the early-age hydration of the cement paste. Specifically, the rate of loss of flowability (or flowability retention in reverse), placement limit, hardening rate, acceleration angle, and yield stress growth rate constant were quantified. The results show that:

- The flowability retention and placement time were significantly enhanced by increasing the content of CA. On the other hand, the hardening rate, acceleration angle, and yield stress growth rate decreased with the increasing CA content. In contrast, an increase in PCE dosage showed a limited effect on the flowability retention and placement time due to the competitive adsorption of the citric acid in lieu of PCE.
- The effect of the PCE dosage was more noticeable on the hardening rate metrics, wherein the hardening rate decreased with the increasing PCE dosage.
- The paste made with 2% CA and 0.1% PCE provided an overall best workability performance in terms of low average yield stress in the flowability regime ($\sigma_{y_{i}}$), low rate of flowability loss ($m_{1}$), moderate placement time ($t_{p}$), and moderate hardening rate metrics ($m_{2},\;\theta_{p}$). However, different applications, such as 3D printing, extended pumping, or oil-well cementing, will feature different combinations of the metrics: $\sigma_{y_{i}}$, $m_{1}$, $t_{p}$, $m_{2},\;\theta_{p}$, and k, for favorable mix design.

Thus, the described rheology-based method can be useful to assess different workability metrics to guide material selection and mixture proportioning for various construction applications, from precast to pumping and additive manufacturing of concrete. Consistent with the existing literature, it is noted that, while a variety of rheology geometry and protocols can be applied to generate the workability metrics that show similar trends as demonstrated in this study, different rheology procedures as well the shear history of the cement paste clearly affect the actual values of the measured quantities. Therefore, the measured rheological quantities are best used in a relative sense to assess mix proportions rather than interpreted as absolute values.

## Figures and Tables

**Table 1:** Oxide composition of CSA cement.

Element	Na_2_O	MgO	CaO	Al_2_O_3_	SiO_2_	P_2_O_5_	SO_3_	K_2_O	TiO_2_	Mn_2_O_3_	Fe_2_O_3_	LOI
% Mass	0.01	0.84	43.59	18.68	10.36	0.43	21.14	0.30	0.47	0.08	2.64	1.46

**Table 2:** Results from static yield stress tests with: (i) parallel plate geometry and (ii) vane rotor and cylinder cup geometry.

**(a)** Parallel plate geometry with a gap of 700 μm				
	**m_1_ (Pa/min)**	**m_2_ (Pa/min)**	**t_p_ (min)**	**θ_p_ (deg.)**
Run 1	3.68	248.88	56.45	89.77
Run 2	3.62	243.90	55.75	89.77
Run 3	3.97	266.66	56.70	89.79
Average	3.76	253.14	56.30	89.77
STDEV	0.18	11.96	0.49	0.01
**(b)** Parallel plate geometry with a gap of 1000 μm				
	**m_1_ (Pa/min)**	**m_2_ (Pa/min)**	**t_p_ min)**	**θ_p_ (deg.)**
Run 1	5.41	127.26	95.0	89.55
Run 2	10.59	110.06	101.0	89.48
Run 3	7.04	122.08	90.0	89.53
Average	7.67	119.79	95.3	89.51
STDEV	2.65	8.82	5.50	0.03
**(c)** Vane rotor and cylinder cup geometry, with a vane rotor bob diameter and length of 28 and 42 mm, and an inner diameter of the cylindrical cup of 30.33 mm.				
	**m_1_ (Pa/min)**	**m_2_ (Pa/min)**	**t_p_ (min)**	**θ_p_ (deg.)**
Run 1	5.682	52.47	57.6	88.91
Run 2	4.371	48.02	57.0	88.81
Run 3	5.654	48.44	51.8	88.82
Average	5.236	49.64	55.5	88.84
STDEV	0.749	2.456	3.19	0.055
**(d)** Parallel plate geometry at a 700 µm gap: with ex situ mixing at 250 rpm prior to loading for each step measurement.				
	**m_1_ (Pa/min)**	**m_2_ (Pa/min)**	**t_p_ (min)**	**θ_p_ (deg.)**
Run 1	−6.916	218.12	49.5	89.73
Run 2	−23.45	267.81	51.5	89.78
Run 3	−16.53	279.77	48.5	89.79
Average	−15.63	255.24	59.8	89.77
STDEV	8.30	32.69	1.53	0.031

**Table 3:** Results from dynamic yield stress and plastic viscosity-based tests, using (i) parallel plate geometry and (ii) vane rotor and cylinder cup geometry.

**(a)** Parallel plate geometry with a gap of 700 μm								
**By dynamic yield stress evolution**					**By plastic viscosity evolution**			
	**m_1_ (Pa/min)**	**m_2_ (Pa/min)**	**t_p_ (min)**	**θ_p_ (deg.)**	**m_1_ (Pa·s/min)**	**m_2_ (Pa·s/min)**	**t_p_ (min)**	**θ_p_ (deg.)**
Run 1	0.219	15.90	100.5	86.40	0.0009	0.0176	50.6	1.008
Run 2	0.384	20.65	94.0	87.22	0.0008	0.0194	48.8	1.111
Run 3	0.254	16.14	96.0	86.45	0.0011	0.0160	49.8	0.917
Average	0.286	17.56	96.8	86.69	0.0009	0.0177	49.7	1.012
STDEV	0.180	2.676	3.32	0.462	0.0002	0.0017	0.90	0.097
**(b)** Vane rotor and cylinder cup geometry, with a vane rotor bob diameter and length of 28 and 42 mm, and an inner diameter of the cylindrical cup of 30.33 mm.								
**By dynamic yield stress evolution**					**By plastic viscosity evolution**			
	**m_1_ (Pa/min)**	**m_2_ (Pa/min)**	**t_p_ (min)**	**θ_p_ (deg.)**	**m_1_ (Pa·s/min)**	**m_2_ (Pa·s/min)**	**t_p_ (min)**	**θ_p_ (deg.)**
Run 1	0.05	19.23	90.3	87.02	0.0018	0.015	45.2	0.867
Run 2	0.04	18.89	97.5	86.96	0.0032	0.011	48.9	0.662
Run 3	0.03	24.10	94.4	87.62	0.0025	0.011	45.0	0.653
Average	0.04	20.74	94.1	87.20	0.0025	0.012	46.4	0.727
STDEV	0.01	2.916	3.61	0.360	0.0007	0.002	2.20	0.121

## Data Availability

Not applicable.
